# Release of Dengue Virus Genome Induced by a Peptide Inhibitor

**DOI:** 10.1371/journal.pone.0050995

**Published:** 2012-11-30

**Authors:** Shee-Mei Lok, Joshua M. Costin, Yancey M. Hrobowski, Andrew R. Hoffmann, Dawne K. Rowe, Petra Kukkaro, Heather Holdaway, Paul Chipman, Krystal A. Fontaine, Michael R. Holbrook, Robert F. Garry, Victor Kostyuchenko, William C. Wimley, Sharon Isern, Michael G. Rossmann, Scott F. Michael

**Affiliations:** 1 Department of Biological Sciences, Purdue University, West Lafayette, Indiana, United States of America; 2 Department of Biological Sciences, Florida Gulf Coast University, Fort Myers, Florida, United States of America; 3 Department of Microbiology and Immunology and Graduate Program in Cellular and Molecular Biology, Tulane University Health Sciences Center, New Orleans, Louisiana, United States of America; 4 Department of Biochemistry, Tulane University Health Sciences Center, New Orleans, Louisiana, United States of America; 5 Department of Pathology, University of Texas Medical Branch, Galveston, Texas, United States of America; 6 Emerging Infectious Diseases, Duke–NUS, Department of Biological Sciences, National University of Singapore, Singapore, Singapore; Utah State University, United States of America

## Abstract

Dengue virus infects approximately 100 million people annually, but there is no available therapeutic treatment. The mimetic peptide, DN59, consists of residues corresponding to the membrane interacting, amphipathic stem region of the dengue virus envelope (E) glycoprotein. This peptide is inhibitory to all four serotypes of dengue virus, as well as other flaviviruses. Cryo-electron microscopy image reconstruction of dengue virus particles incubated with DN59 showed that the virus particles were largely empty, concurrent with the formation of holes at the five-fold vertices. The release of RNA from the viral particle following incubation with DN59 was confirmed by increased sensitivity of the RNA genome to exogenous RNase and separation of the genome from the E protein in a tartrate density gradient. DN59 interacted strongly with synthetic lipid vesicles and caused membrane disruptions, but was found to be non-toxic to mammalian and insect cells. Thus DN59 inhibits flavivirus infectivity by interacting directly with virus particles resulting in release of the genomic RNA.

## Introduction

**Figure 1:**
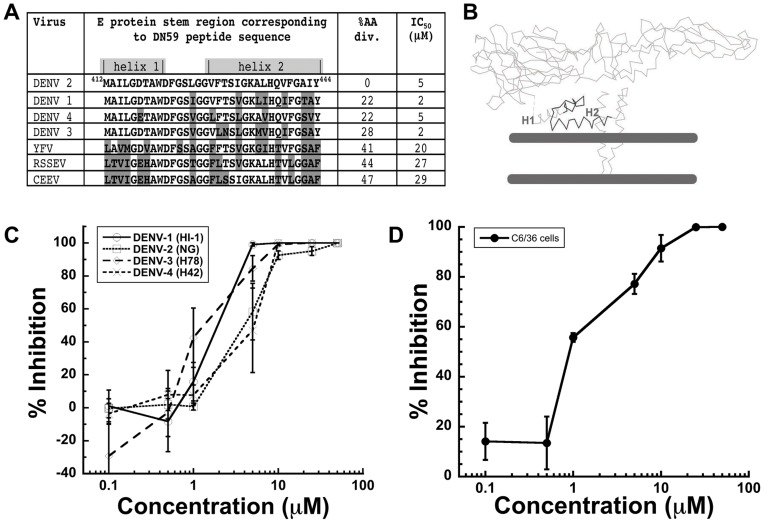
The DN59 peptide inhibits dengue virus infectivity. (A) Sequence comparison of the DN59 amino acid sequence, representing the dengue virus 2 E stem region (residues 412–444), with the stem region of other flaviviruses. YFV - yellow fever virus, RSSEV - Russian spring-summer encephalitis virus, CEEV - Central European encephalitis virus. Non-identical residues are colored in grey. The % amino acid divergence from dengue 2 and IC_50_ values against other flaviviruses are also shown. (B) The C-α backbone of the E protein of dengue 2 as fitted into the 9Å resolution cryoEM map of the mature virus [2]. The region mimicked by DN59 is shown in black outline. Grey bars indicate the lipid bilayer membrane. Part of the stem region helix 2 (H2) interacts with the outer lipid layer of the membrane. (C) FFU reduction assay showing dose response inhibition of infection of dengue virus serotypes 1-4, in mammalian epithelial cells. (D) FFU reduction assay showing dose response inhibition of infection of dengue virus 2 in mosquito cells.

The four dengue virus serotypes, dengue virus types 1, 2, 3 and 4, are major mosquito-transmitted, human pathogens. Currently there are no available vaccines or therapeutics. Dengue is a positive-sense RNA virus, encapsulated by a lipid membrane [1], [2]. The surface of the mature virus particle is composed of 180 envelope (E) glycoprotein molecules and an equal number of membrane (M) protein molecules that assemble at endoplasmic reticulum-derived membranes [1], [2]. The ectodomains of the E glycoproteins are arranged in a herringbone pattern on the surface of the lipid membrane that facilitates binding of the virus to host cells [3] and fusion of the virus with the host membrane after receptor-mediated endocytosis [4], [5], [6]. Each E monomer consists of three domains: DI, DII and DIII [7], [8], [9], [10]. The C-terminal portion of the E protein consists of the stem and membrane anchor regions. The stem region is highly conserved among flaviviruses and is folded into amphipathic helices H1 and H2 that lie underneath the E ectodomain, partially embedded in the lipid envelope (Figure 1A, B) [2].

Ligands that mimic the structure of viral envelope components can sometimes interfere with the normal infection process and, thus, have potential as antiviral agents. For example, the T20 peptide, which is approved for treatment of HIV [11], has a sequence that mimics part of the C-terminal region of the HIV gp41 glycoprotein, and inhibits fusion with host cells [12]. Similarly, DIII of dengue virus E can prevent fusion of virions to host cells [13]. Furthermore, peptides that mimic other regions of E have also been shown to inhibit infection [14], [15]. Some of these peptides bind to E and appear to cause changes in the organization of the glycoproteins on the viral surface [15]. Here we report that a peptide mimicking a highly conserved portion of the E protein stem region causes the release of the genome from the virus particle.

## Results and Discussion

A 33 amino acid peptide, known as DN59, mimics the dengue virus type 2 E stem region (residues 412 to 444). This peptide was previously shown to inhibit the infectivity of dengue 2 virus and West Nile virus, but activity against other flaviviruses and the mechanism of action were unknown [14]. In Figure 1C, we now show that at concentrations of 2-5 µM, the DN59 peptide reduced the infectivity of all four dengue virus serotypes by 50% (IC_50_) in a FFU infection assay using mammalian epithelial cells. The infectivity of other flaviviruses (yellow fever virus, Central European encephalitis virus, and Russian spring-summer encephalitis virus) was inhibited at higher DN59 concentrations (Figure S1A).

**Figure 2:**
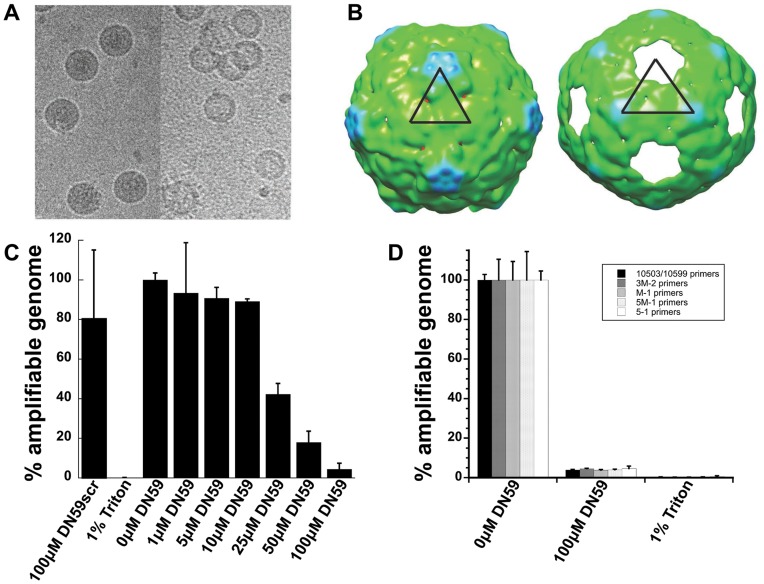
Incubation of mature dengue virus with DN59 peptide results in genome release. (A) CCD images of control dengue virus with 1% (v/v) DMSO (left) and dengue virus incubated with 100 µM DN59 in 1% (v/v) DMSO at 37°C for 30 mins (right). (B) CryoEM image reconstruction of control dengue virus (left) and dengue virus incubated with DN59 (right). Densities are colored according to radius: green (<220Å), cyan (220-230Å), and blue (231-239Å). The icosahedral asymmetric unit is represented by the black triangle. The contour level was chosen as the density that produces a very small hole in the capsid, other than at the five-fold axis. (C) RNase protection assay showing increasing degradation of released viral genome with increasing concentration of DN59. Disruption with detergent (1% triton) resulted in complete degradation. Treatment with a scrambled sequence version of DN59 did not result in significant genome degradation. (D) The RNase protection assay is insensitive to the location of the qRT-PCR primers used to detect the viral genome and indicates that there is no part of the genome that has differential sensitivity to degradation. Bars indicate primer sets targeting different locations in the viral genome.

Cryo-electron (cryoEM) microscopy of dengue virus type 2 particles incubated at 37°C for 30 minutes with 100 µM DN59 in 1% (v/v) DMSO in a 5∶1 molar ratio of peptide to E protein on the virus had lost most of their RNA genomes whereas control virus particles in the presence of 1% (v/v) DMSO showed no visible loss of RNA genome (Figure 2A). Additional images showing larger numbers of control and treated particles are shown in Figure S2. The release of RNA presumably accounted for an increase of viscosity of the virus solution as well as a rather electron dense background on the cryoEM micrographs. Although treatment with peptide may disrupt the symmetry of the virus particle, a three-dimensional icosahedral reconstruction of a small number of particles supported the absence of RNA and suggested the formation of holes at the five-fold vertices through which the RNA might exit (Figure 2B and Figure S3).

The release of viral RNA from the particles was consistent with the results of a genome sensitivity assay conducted by exposing peptide-treated virus particles to RNase digestion, followed by quantitative reverse transcription PCR to determine the amount of protected viral RNA. The RNA genomes of untreated particles were protected from RNase digestion, whereas the genomes of particles co-incubated with increasing concentrations of DN59 were susceptible to digestion in a dose-responsive manner (Figure 2C, D). The peptide concentration required to yield 50% degradation of the genome (17 µM) was approximately four-fold higher than the concentration needed to cause a 50% reduction in infectivity of dengue 2 virus (4.8 µM). This difference might be caused by the use of more than 1,000 times more virus in the genome degradation experiments, or by some treated particles having only partially released genomes after incubation with DN59 (Figure S3A). Although particles with partially released genomes are likely to be non-infectious, their genomes may still have been protected from degradation by RNase. This would cause the IC_50_ for the genome degradation assay to shift upwards in concentration compared to the FFU reduction assay.

**Figure 3:**
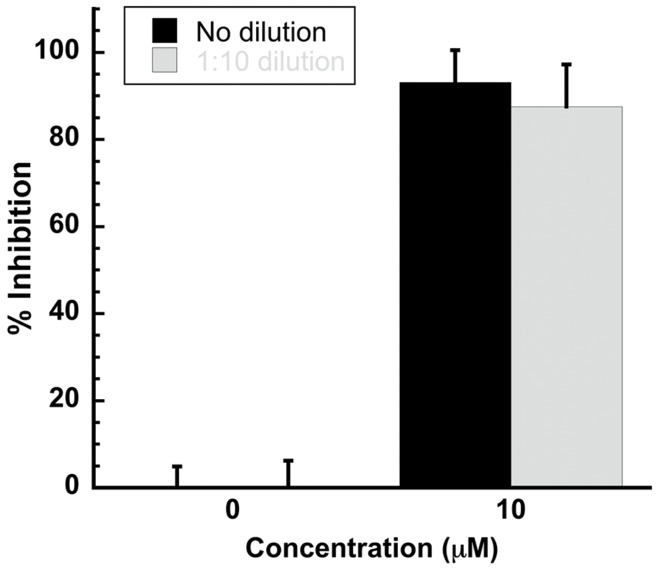
Inhibition of infectivity is not reversible. Dengue virus was incubated with 10 µM DN59, a concentration sufficient to produce approximately 80% inhibition, then either used directly to infect target LLC-MK_2_ cells, or diluted 1∶10 to 1 µM, a concentration that should produce marginal if any inhibition, then used to infect cells. Virus that was treated with 10 µM DN59, then diluted to 1 µM DN59, showed the same level of inhibition of infectivity as virus that was treated and not diluted.

The separation of the genome from the virus particle would be expected to irreversibly destroy infectivity. Reversibility was tested directly by treating virus with peptide at a concentration expected to produce approximately 80% inhibition of infectivity, then diluting the virus:peptide mixture 10 fold to a peptide concentration expected to produce negligible inhibition. No reversibility of inhibition was observed in these experiments (Figure 3).

**Figure 4:**
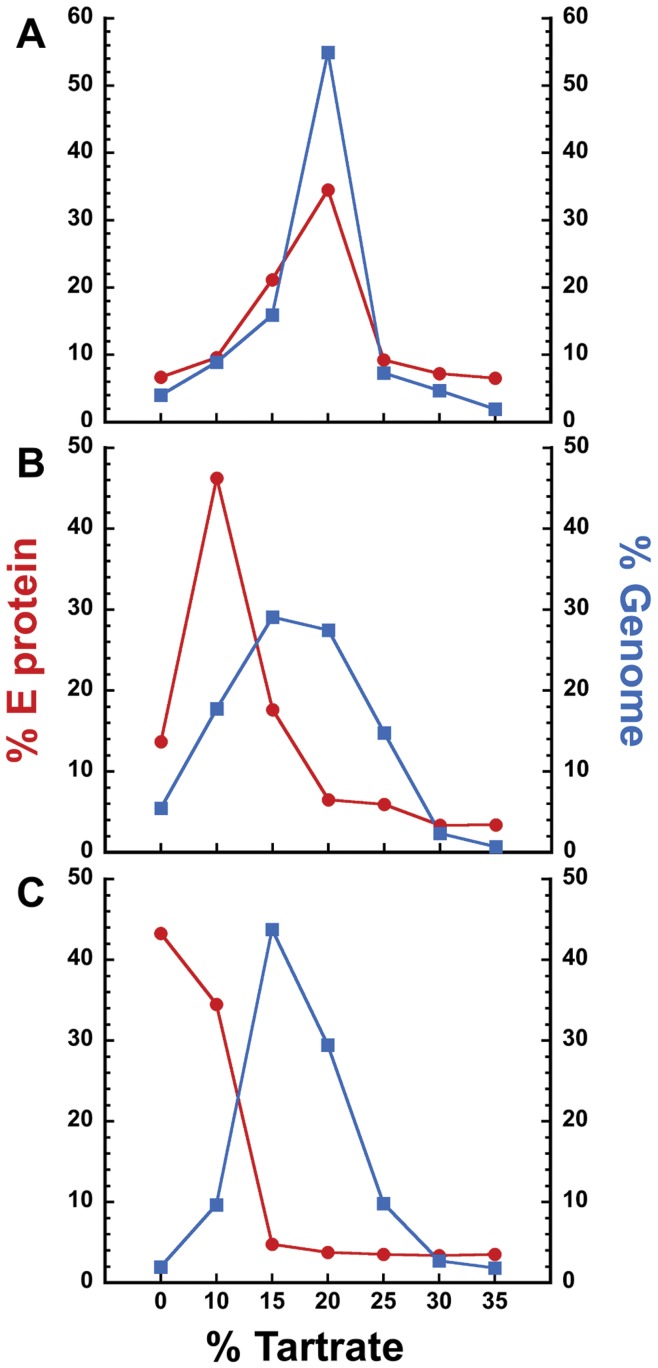
The E protein and genome of virus particles can be separated in a density gradient following treatment with DN59. Dengue virus was untreated (A), treated with 100 µM DN59 (B), or treated with 1% (v/v) triton (C), and centrifuged in a tartrate density gradient. Percent total E protein was measured by ELISA (red circles) and % total genome was measured by qRT-PCR (blue squares) in each fraction. Both peptide treatment and triton detergent treatment result in a separation of E protein and genome in the gradients.

The release of the virus RNA genome was confirmed by centrifuging peptide-treated, untreated, and triton detergent-treated virus particles through a tartrate density gradient, and monitoring the amount of RNA genome and E protein in each fraction. The results showed that the genome and E protein co-migrate in intact virus particles, but migrate to different fractions following peptide or detergent treatment, indicating that the genome and E protein are no longer associated after peptide treatment (Figure 4).

To confirm that there were no other targets for the inhibitory activity of DN59, time of addition and infectivity assays in a different target cell line were conducted. There was no inhibition of infectivity when mammalian target cells were incubated with DN59 and then washed prior to the addition of virus (Figure S1B). Nor was there inhibition of infectivity when DN59 was added after the cells had been infected (Figure S1B). Furthermore, after co-incubation of virus with DN59, infection was inhibited in both mammalian epithelial and mosquito cells (Figure 1C, D), showing that changes of the host cell type and corresponding viral entry pathway did not result in changes of the neutralization profile [16], [17], [18]. Therefore, it can be concluded that DN59 acts directly on the virus particle to release the RNA genome rather than on some other viral or cellular target.

**Figure 5:**
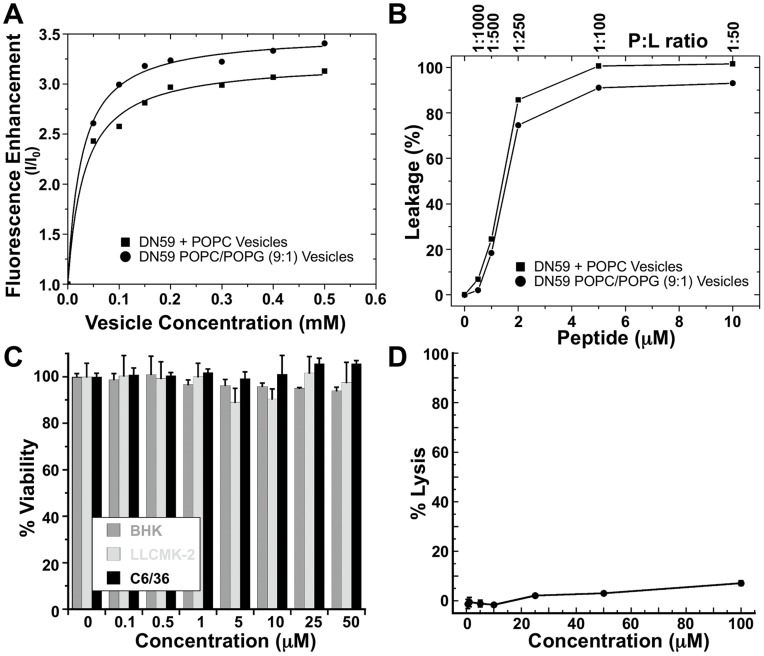
Interaction of DN59 peptide with lipid membranes. (A) DN59 interacts strongly with liposome vesicles. Tryptophan fluorescence-based binding curves for 1 µM DN59 with additions of zwitterionic vesicles made from POPC and anionic vesicles made from POPC and POPG at a 9∶1 ratio. The intensities at 335 nm after each titration are shown and the solid lines are the result of curve fitting with a membrane partitioning equation [34]. (B) DN59 disrupts liposome vesicles. Leakage of the dye/quencher pair ANTS/DPX from 0.5 mM vesicles made from POPC or from POPC/POPG (9∶1). Peptide was added to vesicles and the sample was incubated for 1 hr prior to the measurement of ANTS intensity. Treatment with 10 µM of the highly lytic bee venom peptide melittin was used to achieve 100% leakage. (C) DN59 is not cytotoxic. A mitochondrial reductase metabolic indicator assay (MTT) was used to test the cellular toxicity of DN59 on BHK-21 cells, LLC-MK_2_ cells, and C6/36 cells. There was no significant toxicity of DN59 to cells even at the highest tested concentrations. (D) DN59 is not hemolytic. DN59 was co-incubated with sheep red blood cells and assayed for hemoglobin release. Treatment with 1% (v/v) triton was used to achieve 100% hemolysis.

Based on these experiments, DN59 appears to induce formation of holes in the viral membrane. Thus, DN59 might be expected to interact with lipid membranes and form holes or otherwise disrupt membrane bilayer structures. Consistent with this expectation, a concentration-dependent increase in the fluorescence of the tryptophan residue at peptide position nine was observed when peptide was mixed with liposome vesicles composed of either 1-palmitoyl-2-oleoyl-phosphatidylcholine (POPC), or a 9∶1 molar ratio of POPC and 1-palmitoyl-2-oleoyl-phosphatidylglycerol (POPG), indicative of strong binding (Figure 5A). Also, addition of DN59 peptide to either POPC or POPC/POPG vesicles containing a fluorescent dye and quencher caused extensive disruption of membrane integrity and leakage of contents to occur at concentrations as low as 2 µM (Figure 5B). These observations confirm that DN59 interacts strongly with liposome vesicles and is capable of disrupting artificial lipid bilayers. The observed peptide-lipid membrane interactions are not merely charge based, as binding and disruption occurred with both zwitterionic POPC vesicles as well as negatively-charged 9∶1 POPC/POPG vesicles. Supporting these observations, a recent study of the membrane disruption ability of overlapping peptides from dengue virus type 2 C and E proteins showed that E protein stem derived peptides were highly disruptive to liposomes prepared with a wide variety of lipid compositions [19].

Previously DN59 had been shown to be non-toxic to cultured cells [14]. Similarly, tests using mammalian epithelial and mosquito cells did not show any toxicity at DN59 concentrations as high as 50 µM (Figure 5C). Nor did DN59 induce substantial hemolysis of red blood cells (Figure 5D) illustrating that DN59 does not cause general disruption of cellular plasma membranes at concentrations as high as the 100 µM used for cryoEM. Additionally, DN59 does not inhibit the infectivity of other lipid-enveloped viruses, including Sindbis virus (an alphavirus) [14] or the negative-stranded RNA vesicular stomatitis virus (Figure S1C). The lack of apparent disruption of cellular plasma membranes and other viral membranes may be due to lipid composition, protein incorporation, or active repair of cellular membranes. Dengue virus particles bud from internal endoplasmic reticulum membranes of infected cells and so likely have a different composition from the plasma membrane, although the membrane disruption activity of stem region peptides is not strongly influenced by lipid membrane composition [19].

Schmidt et al. [20], [21] studied a series of similar dengue E protein stem region peptides whose sequences extensively overlap the sequence of DN59 (residues 412-444 of dengue virus type 2 E protein). Consistent with our earlier work [14], they showed that their most active peptide (residues 419 to 447) inhibits dengue virus infection during an entry step and can bind to synthetic lipid vesicles. Furthermore, they reported that their peptide bound to the post-fusion trimeric form of recombinant dengue surface E protein [5], [6] at low pH, but did not bind to the monomeric E protein at neutral pH. They therefore proposed that the peptide neutralizes the virus by first attaching to the viral membrane, and subsequently interacting with the E post-fusion trimers that form when the virus encounters the low pH environment of the endosome, thereby preventing fusion of the virus to the endosomal membrane. Here, however, we have shown that DN59 can induce the formation of holes in the viral membrane, release the genome, and causes the viral particles to become non-infectious even before interacting with cells. The discrepancy in the mechanism of neutralization detected by our group and Schmidt et al. could possibly be due to the differences in peptide concentration used in these assays. Schmidt et al. showed that 1 µM of the peptide could neutralize 2.5×10^4^ infectious virus particles, whereas in our cryoEM studies, the same concentration of DN59 causes RNA release from of 1×10^10^ virus particles. However, direct comparison between these two assays may not be possible. Van der Schaar et al. [22] showed that only a small percentage of the total virus (in the range of 1∶2600 to 1∶72000) is infectious. Since the neutralization test by Schmidt et al. [20] only shows the number of infectious virus particles, the actual total number of virus particles is not known.

The most likely mechanism by which DN59 or other stem region peptides can penetrate the outer layer of E glycoproteins and gain access to the virus membrane is by way of dynamic “breathing” of the virus particle [23], [24], [25], [26]. The ease with which the virus can breathe will depend on the stability of the virus, which may account in part for the differing inhibitory activities against different flaviviruses (Figure S1A). Once the DN59 peptide has inserted itself between the E ectodomain and the membrane, it likely competes with and displaces the virus E protein stem region (helices H1 and H2) for binding to the lipid membrane and the “underside” of the E protein. Formation of holes in the viral membrane large enough for the escape of the RNA genome may involve structural changes in the surface E and M proteins, or may be due to the action of the peptide alone, similar to what is observed for some anti-microbial peptides [27], [28] and what we observed with liposome vesicles. The negative charge on the tightly packaged RNA may also help the RNA to exit the virus particle once the membrane has been destabilized.

Our observations show that DN59, a 33 amino acid peptide mimicking a portion of the dengue virus E protein stem region, functions through an unexpected mechanism that involves disruption of the viral membrane and release of the viral genome.

## Materials and Methods

### Viruses and Cells

Dengue virus 1 (HI-1), dengue virus 2 (NGC-2), dengue virus 3 (H-78), dengue virus 4 (H-42), and yellow fever virus (17-D) were propagated in LLC-MK_2_ cells (American Type Culture Collection (ATCC), Manassas, VA, cat. no. CCL-7) [15]. Russian spring summer encephalitis virus (Sofjin), and Central European encephalitis virus (Hypr) were propagated in BHK-21 cells (ATCC, cat. no. CCL-10). C6/36 cells (ATCC, cat. no. CRL-1660) were maintained in Dulbecco’s modified eagle medium (DMEM) with 10% fetal bovine serum (FBS), 100 µM Non-essential amino acids, 2 mM Glutamax, 100 U/ml penicillin G, 100 µg/ml streptomycin and 0.25 µg/ml amphotericin B, at 30°C with 5% CO_2_. For the cryo-electron microscopy studies, dengue virus 2 (16681) was grown in C6/36 cells and the tissue culture supernatant was collected on day 3-4, spun at 2,704×*g* for 10 minutes at 4°C. 8% PEG in NTE (120 mM NaCl, 12 mM Tris, pH 8.0, 1 mM EDTA) was added to the tissue culture supernatant and mixed. The solution was then allowed to sit overnight before the PEG precipitated virus was centrifuged at 14,636×*g* for 1 hr. The pellet was resuspended in 1 ml NTE buffer, loaded onto a 24% (w/v) sucrose cushion and centrifuged at 175,587×*g* for 90 min. Pellets were resuspended overnight in NTE before being loaded onto a 10-30% (w/v) potassium sodium tartrate step gradient and centrifuged at 175,587×*g* for 2 hrs. Purified virus was collected from the 20% potassium-tartrate fraction. The virus solution was then buffer exchanged to NTE buffer using an Amicon Ultra-4 centrifugal filter.

### Peptides

Peptide stocks of DN59, the 33 amino acid pre-anchor domain peptide (MAILGDTAWDFGSLGGVFTSIGKALHQVFGAIY) and a randomly scrambled version of the peptide, DN59scr (YFIDTSGAIWGASHLTGVLFDFMGIQGGAVLAK) were purified and then prepared as approximately 1 mM stocks in 20% dimethylsulfoxide (DMSO): 80% H_2_O [15]. Concentrations were calculated from side chain absorbance at 280 nm.

### Focus Forming Unit (FFU) Reduction Assay

FFU reduction assays were performed as previously described [14]. Approximately 200 FFU of virus were incubated with peptide in serum-free DMEM for 1 hr at room temperature before infecting LLC-MK_2_ cell monolayers for 1 hr at 37°C, and overlaying with media containing 0.85% (w/v) Sea-Plaque Agarose (Cambrex Bio Science, Rockland, ME). Infected cells were incubated at 37°C with 5% CO_2_ for 2 days (yellow fever virus), 3 days (dengue virus 3 and 4, Russian spring summer encephalitis virus and Central European encephalitis virus) or 5 days (dengue virus 1and 2). Infected cultures were fixed with 10% (v/v) formalin, permeablized with 70% (v/v) ethanol, and foci were detected using mouse monoclonal antibodies against yellow fever virus (Chemicon, Temecula, CA), dengue (E60), or polyclonal anti-Kumlinge virus rED3 antisera, followed by horseradish peroxidase-conjugated goat anti-mouse immunoglobulin (Pierce, Rockford, IL), and developed using AEC chromogen substrate (Dako, Carpinteria, CA) as previously described [15], [29].

### Virus Inhibition on C6/36 Cells

C6/36 monolayers were infected with approximately 7,600 FFU of dengue virus 2 at 37°C for 1 hr before being aspirated, complete culture media added, and incubated at 37°C and 5% CO_2_. After 72 hrs, RNA was isolated from cells using an RNeasy Mini Kit (Qiagen, Valencia, CA). qRT-PCR was performed as previously described [18].

### Cryo-electron Microscopy

1 mM DN59 in 10% (v/v) DMSO was mixed with 18 µl of mature dengue virus to give a final DN59 concentration of 100 µM with 1% (v/v) DMSO. The mixture was incubated at 37°C for 30 min, then 4°C for 2 hrs and frozen on holey carbon grids. Dengue virus without peptide and dengue virus incubated with DMSO only controls were also frozen. Images were collected with a Philips CM200 cryo-electron microscope using 200 KV, a magnification of 50,000, an electron dose of 25 e^−/^A^2^, and taken at about 4.3 to 7 µm out-of-focus. Thirty-eight DN59 treated dengue virus particles were selected for three-dimensional (3D) image reconstruction. Initial models for 3D reconstructions were generated using the program starticos in EMAN [30]. This program correlates each image with itself after rotating by 72°, 120° and the starting model is essentially a random model based on combining the three orientations related by icosahedral symmetry. Subsequently, thirty iterations were performed in which the orientation of each of the raw images was determined relative to the current model from the previous cycle using the program SPIDER [31]. The images were split into two groups for resolution estimation, by observing the point at which the Fourier shell coefficient fell below 0.5 [32]. The final resolution was about 40 Å no matter whether N was chosen to be 3, 5, 8, 10 or 12 (Figure S3). Contours were chosen to only just avoid opening a hole in the capsid other than at the five-fold vertices.

### RNase Assay and qRT-PCR

Approximately 1.4-2.9×10^4^ FFU of dengue virus 2 was incubated with DN59 for 1 hr at room temperature and then digested with micrococcal nuclease (New England BioLabs, Ipswich, MA) for 1 hr at 37°C. RNA was extracted using the Qiagen RNeasy mini kit and qRT-PCR was performed as above using 10503F/10599R [33], 3 M-2F (TCACCAAATCCCACGGTAGAAGCA)/3 M-2R (AGGGCATGTATGGGTTGAGAACCT), M-1F (GAGGCTGGAAGCTAGAAG)/M-1R (GAGATACGGCACCTATGG), 5 M-1F (AAGCAGAACCTCCATTCGGAGACA)/5 M-1R (AAACACTCCTCCCAGGGATCCAAA), and 5-1F (AATCCCACCAACAGCAGGGATACT)/5-1R (CGCCATCACTGTTGGAATCAGCAT) primer sets.

### Infectivity Inhibition Reversibility Assay

Similar to the FFU reduction assays, approximately 200 FFU of dengue virus 2 were incubated with 0 or 10 µM DN59 in a total volume of 100 µl serum-free DMEM for 1 hr at room temperature. Immediately before infecting LLC-MK_2_ cell monolayers, the virus/peptide mixtures were diluted with serum-free DMEM to 1 ml, reducing the concentration of DN59 to 1 µM.

### Tartrate Density Gradient Assay

Approximately 10^6^ FFU of dengue virus 2 produced in LLC-MK_2_ cells and purified as described above for the cryo-electron microscopy studies, was treated with 100 µM DN59 or 1% (v/v) triton X-100 for 30 min at 37°C. Treated virus was loaded onto a 10-35% (w/v) potassium sodium tartrate step gradient and centrifuged at 175,117×*g* for 2 hrs. Individual fractions were collected and assayed for virus genome and E protein. Genome quantitation was carried out by qRT-PCR as described above for the RNase sensitivity assay using the 10503F/10599R primer set [33]. E protein detection was carried out using modified ELISA. High bind 96-well plates (Costar, Corning, NY) were coated with concavalin A (Vector Laboratories, Burlingame, CA) at 25 mg/ml in 0.01 M HEPES (for 1 hr and washed with PBS containing 0.1% (v/v) Tween-20. Equal aliquots of each gradient fraction were added for 1 hr to allow binding of E to the concavalin A and then washed again. Captured E protein was detected using a human anti-E monoclonal antibody, followed by goat anti-human HRP conjugate. After a final wash, color was developed with tetramethylbenzidine-peroxide (TMB)-H_2_O_2_ stopped by adding 1% (v/v) phosphoric acid. Optical density was measured at 450 nm.

### Lipid Vesicle Binding by Tryptophan Fluorescence

The lipids 1-palmitoyl-2-oleoyl-phosphatidylcholine (POPC) and 1-palmitoyl-2-oleoyl-phosphatidylglycerol (POPG) were purchased from Avanti Polar Lipids (Alabaster, AL). Lipids in chloroform solution were dried under vacuum overnight followed by hydration with phosphate buffered saline (PBS). Ten cycles of freezing and thawing were used to ensure solute entrapment. Unilamellar vesicles of 0.1 µm diameter were made by extrusion of the lipid suspension through 0.1 µm polycarbonate filters [34]. Tryptophan fluorescence spectra were measured on an SLM-Aminco fluorescence spectrophotometer. Samples were mixed in a 10×4 mm quartz cuvette and spectra were collected with excitation at 270 nm and emission from 300-450 nm. Lipid titrations were made from a 50 mM stock solution of vesicles in PBS, with 15 minutes of equilibration after each titration before measurements were made. Binding curves were obtained by taking the intensity at 335 nm for each spectra, minus the intensity of the appropriate peptide-free control sample.

### Liposome Vesicle Leakage

The fluorescent dye 8-aminonaphthalene-1,3,6-trisulfonic acid (ANTS) and its obligate quencher p-xylene-bis-pyridinium bromide (DPX) were purchased from Invitrogen (Carlsbad, CA). Vesicles were prepared with ANTS/DPX entrapped inside where DPX quenches ANTS fluorescence [34]. Lipids were hydrated with buffer containing 50 mM ANTS and 12.5 mM DPX followed by extrusion and then gel filtration chromatography using Sephadex G-200 to exchange the external ANTS/DPX solution for buffer. In leakage experiments, 0.5 mM vesicles were mixed with peptide from 0.5 to 10 µM to give peptide to lipid ratios ranging from 1∶50 to 1∶1000. The increase in ANTS fluorescence after 1 hr incubation with peptide reports on vesicle leakage. A complete leakage control was achieved by the addition of 10 µM of the lytic bee venom peptide melittin.

### Cell Toxicity Assays

Cytotoxicity of DN59 was measured by mitochondrial reductase activity using the TACS™ MTT cell proliferation assay (R&D Systems Inc., Minneapolis. MN). DN59 in serum-free DMEM was added to LLC-MK_2_, BHK, or C6/36 cells for 1 hr at 37°C, the solution was removed and the cells incubated at 37°C in complete medium with 5% CO_2_ for 24 hrs.

### Hemoglobin Release Assay

Sheep red blood cells (RBC) (Lampire Biological Products, Pipersville, PA) in anti-coagulant K2-EDTA, were washed and resuspended in PBS to a final concentration of 10% (v/v). Peptide was added to 2% RBC, incubated at 37°C for 1 hr and centrifuged at 13,000 rpm. Supernatants were collected and the absorbance at 560 nm was measured. Results were normalized against treatment with 1% (v/v) triton X-100 as a control for 100% hemolysis.

### Vesicular Stomatitis Virus Plaque Reduction Assays

Plaque reduction assays in LLC-MK_2_ cells were carried out in a similar manner as above, except that a 1.2% solution of methylcellulose (FMC, Philadelphia, PA) in complete medium was used in place of agarose. Vesicular stomatitis virus eGFP-P was incubated for 24 hrs at 37°C before overlays were aspirated, rinsed with PBS, and plaques were visualized for GFP expression [35].

## Supporting Information

Figure S1 **Inhibitory effect of DN59 is dependent on its interaction with flavivirus particles.** (A) Co-incubation of DN59 with other flaviviruses showed dose response inhibition in a focus-forming unit reduction assay with somewhat higher 50% inhibition concentrations compared to dengue virus. (B) Focus-forming unit reduction assay indicates that DN59 has no inhibitory effect on dengue virus infection when the peptide is added to LLCMK-2 cells and removed prior to the addition of dengue virus, or when DN59 is added to cells that had already been infected. (C) DN59 was co-incubated with the enveloped, negative-stranded RNA, vesicular stomatitis virus (VSV), and infectivity was assayed in a plaque reduction assay. No substantial inhibition of VSV was observed.
(TIF)

Figure S2 **Homogeneity of virus particle preparations used for EM imaging.** Lower magnification CCD images of control (A) and DN59 treated (B) dengue virus showed that the control virus particles were relatively homogenous and mature. DN59 treated particles clumped and were obscured by an electron dense material.
(TIF)

Figure S3 **CryoEM image reconstruction of DN59 treated dengue 2 virus.** (A) CryoEM image of DN59 treated particles. The particles appeared empty. (B) Reconstruction and validation of the cryoEM structure. Different starting models (West Nile virus, as well as five reference free models generated using the program starticos [30] with a different number (N) of particles used in the classification of particles with five-fold, three-fold and two-fold projected views) were used to reconstruct untreated control and DN59-treated dengue particles. To permit a direct comparison of the reconstructions produced by these different starting models, the arbitrary contour levels of the control maps were set at two different values. The high contour level was adjusted until the five-fold densities were just visible and the lower contour level was adjusted until holes at the three-fold vertices were just visible. For the DN59-treated dengue virus particles, the contour level was adjusted until the holes at three-fold vertices were just visible. Five out of six starting models for cryoEM image reconstruction of DN59-treated dengue virus had a dominant hole at the five-fold vertices. None of the untreated dengue virus controls had a hole at the five-fold vertices.
(TIF)
