# Supplementary figures and images for: Release of Dengue Virus Genome Induced by a Peptide Inhibitor
Source: PLoS One. 2012 Nov 30;7(11):e50995. doi: 10.1371/journal.pone.0050995 (PMC3511436; doi:10.1371/journal.pone.0050995)

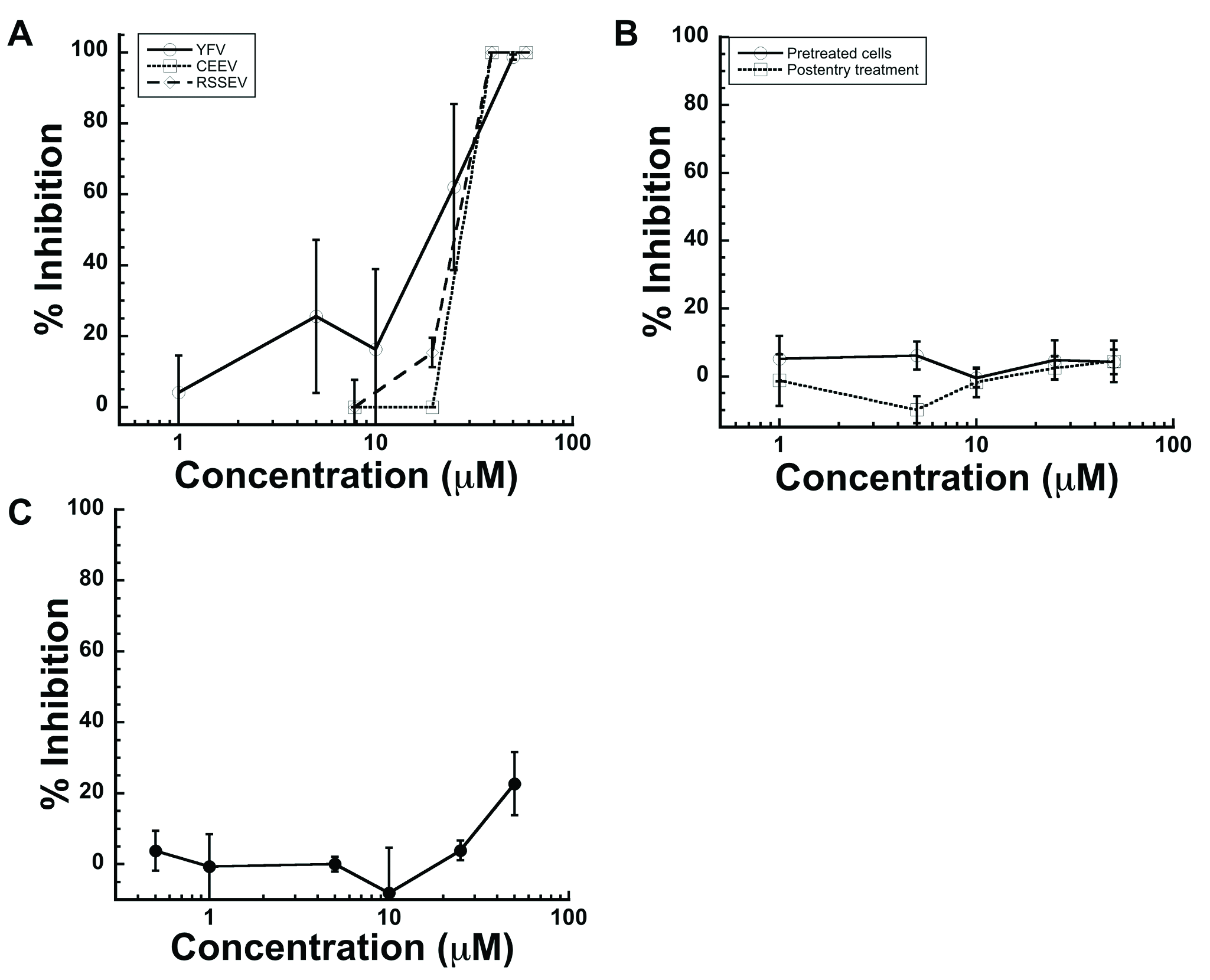

Supplement: Figure S1 — Inhibitory effect of DN59 is dependent on its interaction with flavivirus particles. (A) Co-incubation of DN59 with other flaviviruses showed dose response inhibition in a focus-forming unit reduction assay with somewhat higher 50% inhibition concentrations compared to dengue virus. (B) Focus-forming unit reduction assay indicates that DN59 has no inhibitory effect on dengue virus infection when the peptide is added to LLCMK-2 cells and removed prior to the addition of dengue virus, or when DN59 is added to cells that had already been infected. (C) DN59 was co-incubated with the enveloped, negative-stranded RNA, vesicular stomatitis virus (VSV), and infectivity was assayed in a plaque reduction assay. No substantial inhibition of VSV was observed. (TIF) [file pone.0050995.s001.tif]

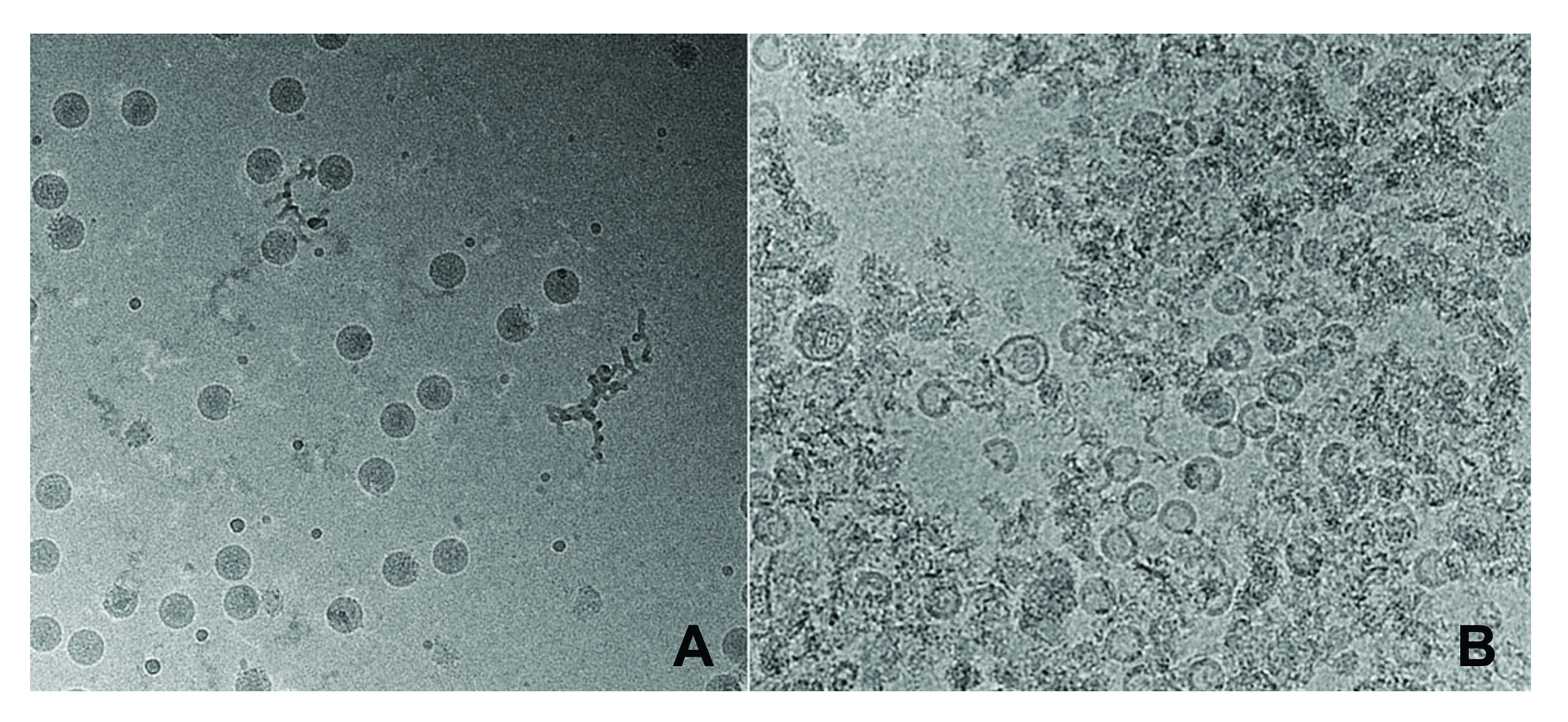

Supplement: Figure S2 — Homogeneity of virus particle preparations used for EM imaging. Lower magnification CCD images of control (A) and DN59 treated (B) dengue virus showed that the control virus particles were relatively homogenous and mature. DN59 treated particles clumped and were obscured by an electron dense material. (TIF) [file pone.0050995.s002.tif]

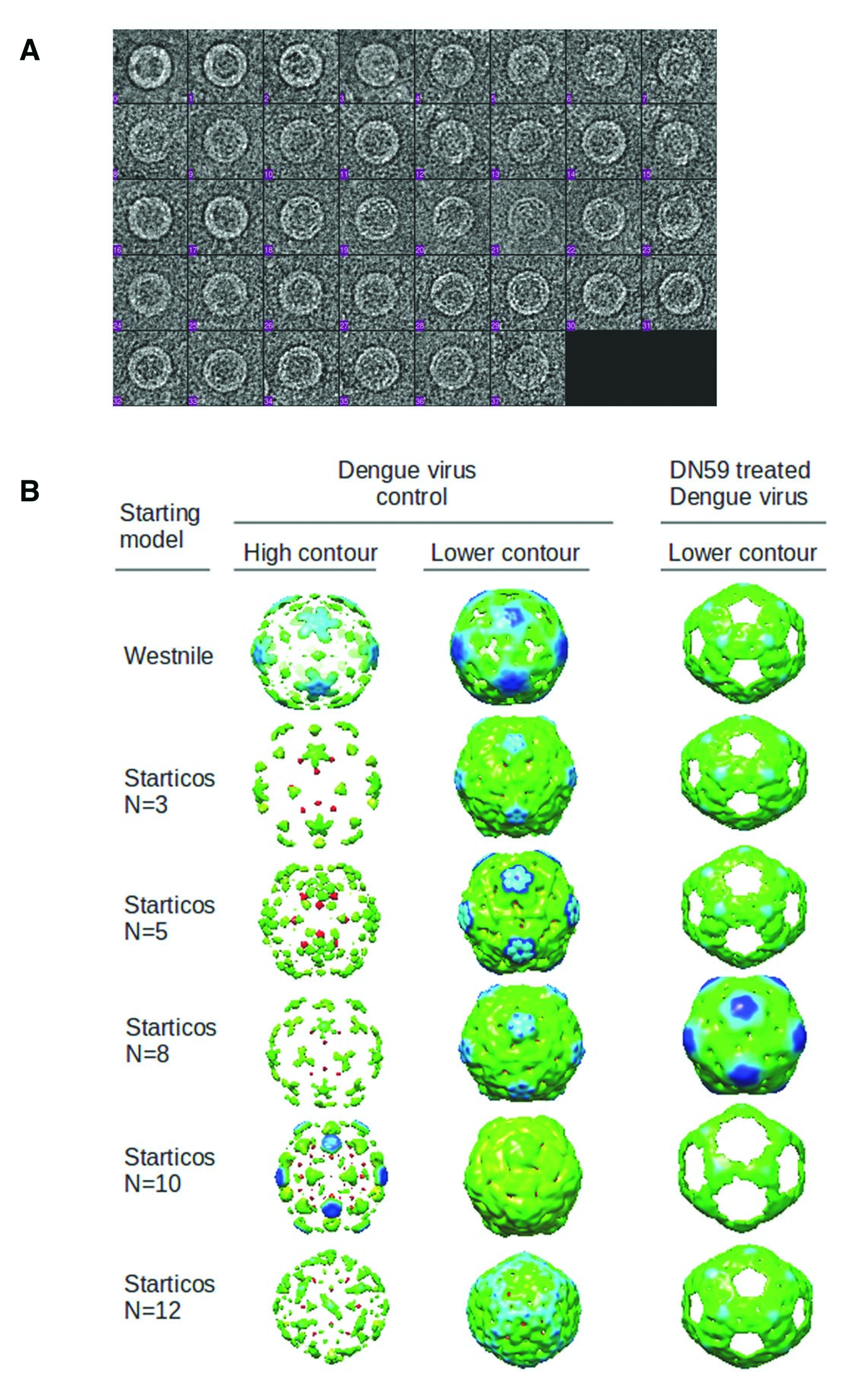

Supplement: Figure S3 — CryoEM image reconstruction of DN59 treated dengue 2 virus. (A) CryoEM image of DN59 treated particles. The particles appeared empty. (B) Reconstruction and validation of the cryoEM structure. Different starting models (West Nile virus, as well as five reference free models generated using the program starticos [30] with a different number (N) of particles used in the classification of particles with five-fold, three-fold and two-fold projected views) were used to reconstruct untreated control and DN59-treated dengue particles. To permit a direct comparison of the reconstructions produced by these different starting models, the arbitrary contour levels of the control maps were set at two different values. The high contour level was adjusted until the five-fold densities were just visible and the lower contour level was adjusted until holes at the three-fold vertices were just visible. For the DN59-treated dengue virus particles, the contour level was adjusted until the holes at three-fold vertices were just visible. Five out of six starting models for cryoEM image reconstruction of DN59-treated dengue virus had a dominant hole at the five-fold vertices. None of the untreated dengue virus controls had a hole at the five-fold vertices. (TIF) [file pone.0050995.s003.tif]
